# Macrophage Immunometabolism and Inflammaging: Roles of Mitochondrial Dysfunction, Cellular Senescence, CD38, and NAD

**DOI:** 10.20900/immunometab20200026

**Published:** 2020-07-01

**Authors:** Johnathan R. Yarbro, Russell S. Emmons, Brandt D. Pence

**Affiliations:** 1 School of Health Studies, University of Memphis, Memphis, TN 38152, USA; 2 Bioinformatics Program, University of Memphis, Memphis, TN 38152, USA; 3 Center for Nutraceutical and Dietary Supplement Research, University of Memphis, Memphis, TN 38152, USA

**Keywords:** aging, macrophage, monocyte, NAD, mitochondria, inflammaging, immunometabolism, senescence, SASP, CD38

## Abstract

Aging is a complex process that involves dysfunction on multiple levels, all of which seem to converge on inflammation. Macrophages are intimately involved in initiating and resolving inflammation, and their dysregulation with age is a primary contributor to inflammaging—a state of chronic, low-grade inflammation that develops during aging. Among the age-related changes that occur to macrophages are a heightened state of basal inflammation and diminished or hyperactive inflammatory responses, which seem to be driven by metabolic-dependent epigenetic changes. In this review article we provide a brief overview of mitochondrial functions and age-related changes that occur to macrophages, with an emphasis on how the inflammaging environment, senescence, and NAD decline can affect their metabolism, promote dysregulation, and contribute to inflammaging and age-related pathologies.

## INTRODUCTION

Aging is the highest risk factor for the majority of chronic diseases—including cardiovascular disease, diabetes, stroke, and cancer [1], and a staggering 73% of all deaths worldwide in 2017 were attributable to chronic diseases [2]. Underlying most age-related pathologies is a sterile, chronic, systemic inflammatory state (inflammaging), which is likely a result of a host of factors that become dysregulated with age [3,4]. These “hallmarks of aging” include mitochondrial dysfunction, altered metabolic signaling, defective autophagy and mitophagy, dysbiosis, diminished proteostasis, stem cell exhaustion, telomere attrition, epigenetic changes, genomic instability, and cellular and immune senescence [1,3,5]. These hallmarks are interconnected and co-occur with one another, but all converge on inflammation, as an impairment in any of these can promote inflammation and affect the other hallmarks [5]. The causal driver of inflammaging appears to be an accumulation of damage over time, alongside the gradual decrease in the body’s ability to repair from damage and maintain homeostasis [5,6]. Damaged cells, cell debris, and misfolded/misplaced molecules (garbage) can be sensed by pattern recognition receptors (PRRs) of the innate immune system as damage-associated molecular patterns (DAMPs) and promote inflammation. Inflammaging and dysbiosis drive intestinal permeability resulting in increased circulating levels of bacterial products which further stimulate PRRs via their pathogen-associated molecular patterns (PAMPs) [7]. Chronic stimulation of PRRs and cytokine receptors by DAMPs, PAMPs, and inflammatory cytokines plays a major role in the development of inflammaging, and macrophages are central in this phenomenon [6]. Macrophages play a critical role in removing garbage and bacteria, maintaining homeostasis, and regulating inflammation, and undergo various changes with age which can contribute to age-related pathologies, largely by increasing inflammation. Several studies have demonstrated that macrophages can actively drive age-related pathologies, as depleting macrophages led to diminished inflammatory responses and improved survival outcomes [8–11].

## MACROPHAGE POLARIZATION

Macrophages exhibit exceptional plasticity and can adjust their phenotype in response to various signals in their environment. Traditionally, they have been classified as resting (M0), classically activated (M1), or alternatively activated (M2). Macrophages are generally polarized in vitro to a M1 phenotype using lipopolysaccharide (LPS), interferon-gamma (IFNγ), tumor necrosis factor alpha (TNFα) and/or other Toll-like receptor (TLR) ligands [12]. M1 macrophages have primarily been shown to be catabolic, pro-inflammatory, glycolytic, and bactericidal. They secrete a variety of inflammatory/bactericidal mediators including IL-6, TNFα, IL-1β, IL-12 and reactive oxygen species (ROS) [12], all of which increase in circulation with age [13,14]. M2 macrophages represent the opposite end of the polarization spectrum from M1 macrophages. They rely mainly on oxidative phosphorylation (OXPHOS) and fatty acid oxidation (FAO) for energy, are generally anti-inflammatory, and are involved in tissue repair, angiogenesis, and phagocytosis [15]. This binary classification of macrophage phenotypes is still commonly used for simplification but is inaccurate as macrophage phenotypes exist in vivo across a wider spectrum [15,16].

The local microenvironment plays a critical role in shaping what genes are expressed [17], and macrophages can display both M1 and M2 markers in vivo and are not necessarily exclusively pro-inflammatory or anti-inflammatory [15]. For instance, there is evidence that M2-like macrophages can be pro-inflammatory [18], and that a pro-inflammatory M2 phenotype seems to accumulate in some, but not all, tissues with age [19–24]. M2 phenotypes have recently been divided into M2a, M2b, M2c, and other subdivisions by some authors based off the stimuli used to polarize them in vitro [15]. Traditionally, they have been polarized with IL-4 and IL-13 to the M2a subdivision and most studies generally refer to this subtype when mentioning M2 macrophages. Since macrophages are heterogeneous and the various phenotypes are still being characterized, it is currently unknown exactly how the macrophage phenotypic landscape changes with age, but new metrics from various omics technologies may be able to help elucidate this in the near future [25]. Regardless, it does seem that macrophages increase in number in most, but not all, studied tissue types with age and display an altered physiology that often contribute to pathology [19,22–24,26,27]. Several recent review articles describe these changes in detail [28–30], so we will only briefly mention some of the major changes here which are relevant to this discussion.

## AGE-RELATED MACROPHAGE DYSFUNCTION

Among the age-related changes that occurs to macrophages is a decline in phagocytic ability which has been observed in multiple tissues including the peritoneum [31], lungs [32], bone marrow [33] and brain [34]. This dysfunction could be a consequence of several different factors including senescence [35], defective autophagy [36], reduced NAD availability [37], and impairments in mitochondrial functions such as reduced ATP production, mitochondrial membrane potential (ΔΨm), and increased reactive oxygen species (ROS) production [38,39]. Age-related alterations in macrophage phenotypes may also contribute, as M2-like macrophages are generally more phagocytic and are reduced in some tissues. For example, a recent study in Alzheimer’s patients reported an increase in M1 and a decline in the M2b phenotype, which is highly phagocytic [22]. Aging has also been shown to cause an increased number of bone marrow-derived macrophages (BMDMs) that are skewed towards a M1 phenotype and display impaired phagocytosis and cytokine production [24]. Extrinsic factors in the aging microenvironment likely also play a role, as peritoneal macrophages from young mice injected into the peritoneum of aged mice exhibited impaired phagocytic capacity [31]. It is likely that a combination of all these factors, as well as other aging hallmarks, contribute to phagocytic decline.

Many studies have reported altered TLR expression and cytokine production in macrophages as a result of aging. Macrophages from aged individuals generally exhibit increased basal inflammation and exist in a sustained activated state, likely as a result of chronic stimulation by the inflammaging environment [40]. Often accompanying this low-grade basal inflammation is a state of immune paralysis in which effector responses such as phagocytosis, antigen presentation, and wound healing are impaired [40]. There has been a number of contradictory findings reported in the literature with regards to cytokine production following stimulation in aged macrophages, which may be explained by differing study designs, tissue site of origin [41], or other factors. Several studies have shown increased cytokine, ROS, and/or nitric oxide production following stimulation in aged macrophages [8,42–44]. This may be partially due to an epigenetic rewiring process known as trained immunity, in which macrophages and other innate immune cells exhibit enhanced responsiveness to stimuli they have previously encountered [45]. Trained immunity requires a shift in metabolism towards a glycolytic state [46], as it promotes cholesterol synthesis, fumarate accumulation, and glutaminolysis which mediates epigenetic reprogramming [47]. This reprogramming is characterized by trimethylation of lysine 4 at histone 3 (H3K4me3) and acetylation of lysine 27 (H3K27ac) on the promotor region of genes involved in immune signaling and metabolism. This results in increased expression of proinflammatory genes such as IL-6, TNFα, MCP-1, as well as key glycolytic genes such as hexokinase-2 and phosphofructokinase [48].

It has been proposed that lower doses of PAMPs and DAMPs may induce trained immunity, while higher doses can lead to an opposite effect known as immune tolerance [49]. Immune tolerance is characterized by a diminished responsiveness to stimuli, usually as a result of receptor desensitization [45], which has been demonstrated for various TLRs in macrophages [50]. In contrast to the studies mentioned above, several studies have shown that macrophages and monocytes have decreased responsiveness to stimuli [51–55], and these disparate findings could possibly be due to differences in the epigenetic state when analyzed. Immune tolerance and trained immunity have not been well studied in the context of aging, but it is likely that these epigenetic processes play a role in inflammaging. Aging involves a complex reshaping of the immune system, and persistent stimulation by DAMPs and PAMPs may drive non-uniform changes which cause some cellular responses to be impaired, some to be preserved, and others to be hyper-activated. As these epigenetic changes are largely dependent on bioenergetic processes, dysfunctional mitochondria and the resulting metabolic derangements may be central to this phenomenon. When PRR stimulation is relatively low, immune cells can efficiently respond by altering their metabolism to maintain adequate production of the necessary biomolecules needed to perform their functions. For instance, upregulating glycolysis to meet the energy demand for effective cytokine and ROS production to kill invading pathogens. Rising PRR stimulation and excessive oxidative stress that occur with aging may induce bioenergetic defects, as the cell cannot keep up with the energy demand, which dysregulates trained immunity and immune tolerance and may contribute to immunosenescence [49]. This has been demonstrated in BMDMs from aged mice which exhibited dysregulated immune tolerance due to an inability to shift from OXPHOS to glycolysis following LPS stimulation [51]. NAD decline with age, which is described in a later section of this review, likely contributes to these defects. NAD-dependent sirtuins play essential roles in regulating metabolic functions and were shown to be involved in initiating and resolving immune tolerance in monocytes via metabolic reprogramming [56,57]. How this precisely contributes to age-related epigenetic changes and macrophage dysfunction has yet to be determined, but it is clear that metabolic dysregulation and mitochondrial impairments play an important role.

## MITOCHONDRIA AND AGING

Mitochondria lie as the central hub for cellular metabolism. Historically, mitochondria have been viewed in the context of ATP generation as the site for the citric acid (TCA) cycle and OXPHOS, however; mounting evidence highlights their role as signaling propagators either through the release of proteins, metabolites, and ROS, or as a scaffold for signaling complexes [58]. Mitochondrial dysfunction contributes to inflammaging and immunosenescence and has been linked to a myriad of diseases including cardiovascular disease, cancer, metabolic diseases, and aging [59–62]. The underlying mechanism by which aging occurs is not fully understood, although many hypotheses have been ventured. Denham Harman put forth the free radical theory of aging in 1956, briefly summarized as the cumulative damage from ROS to DNA, lipids, and proteins which drives aging and age-related diseases [63]. Challenges to this paradigm have arisen with the observations of ROS in normal physiological processes, beneficial hormetic adaptions resulting from low grade exposure to ROS such as with exercise, and observed dysfunction in other organelles including the endoplasmic reticulum (ER) and lysosomes resulting in misfolded proteins [64–66]. Nevertheless, a mounting body of evidence have coalesced to highlight a relationship between aging and mitochondrial health.

Inflammatory activation of the immune system can be triggered through sensing by PRR of DAMPs or PAMPs. PRRs constitute a wide range of receptors including NOD-like receptors (NLRs), receptors for advanced glycation end-products (RAGEs), and TLRs that affect inflammatory responses. For instance, mitochondrial DNA (mtDNA), which shares many similarities with bacterial DNA due to their shared prokaryotic origins, can serve as an inflammatory stimulus by acting as a DAMP. The mitochondrial proteome is maintained by mtDNA, distinct from the nuclear genome. mtDNA is vulnerable to mutation and lesion from the hazardous ROS-rich mitochondrial environment as it lacks the protective histone structures of nuclear DNA. Despite this, mtDNA remains functional in the presence of high degrees of mutation load of 60–90% before defects in OXPHOS manifest [67–69]. However, mtDNA is released from apoptotic and stressed cells [70,71] and is a potent DAMP, activating innate immune cells in a TLR9 [72], NLRP3 inflammasome [73], and cGAS/STING dependent fashion [74]. Indeed, mtDNA has been observed to stimulate immune activation following traumatic injury [72] and heart failure [75]. During aging, mtDNA has been observed to increase in tissues and circulation and correlates with inflammatory markers; further, mtDNA was shown to promote proinflammatory cytokine production in monocytes [76,77]. Understanding of mitochondrial structure and function may shed light on their contribution to inflammaging.

## MITOCHONDRIAL STRUCTURE AND FUNCTION

Mitochondria are dynamic organelles, translocating around the cell and forming interconnected networks. Maintenance, transcription, and packaging of mtDNA is controlled by mitochondrial transcription factor A (TFAM) [78,79]. Mitochondria possess a double membrane, containing an outer mitochondrial membrane (OMM) and invaginated inner mitochondrial membrane (IMM) composed of cardiolipin to form cristae where the OXPHOS machinery is distributed throughout [80]. The electron transport chain (ETC) is composed of a series of 4 protein complexes, CI-CIV, on the lateral IMM. The final complex, F1F0 ATP synthase is the primary generator of cellular ATP. Briefly, NADH and FADH2 are reduced to NAD^+^ and FAD respectively by a series of redox reactions at complexes I-IV, whereby protons are pumped into the intramembrane space to create the electrochemical gradient ΔΨm (further reviewed [81]). F1F0 ATP synthase uses the ΔΨm to catalyze ATP generation from ADP. Besides fueling F1F0 ATP synthase, the ΔΨm serves as a buffer for Ca^2+^ and is an effective measure of mitochondrial quality. Maintenance of the ΔΨm is necessary for driving OXPHOS, maintaining the intramembrane space, and for cellular homeostasis. Interestingly, the ΔΨm has recently been observed to be heterogenous between cristae throughout mitochondria [80]. The varying ΔΨm throughout the mitochondria may serve to allow for different aspects to focus on various functions such as OXPHOS vs ROS generation. Similarly, it may serve as a protective mechanism to highlight damaged areas or prevent the spread of damage in defective OXPHOS.

At the mitochondrial level, divergent stimuli elicit unique utilization of mitochondria to carry out cellular response. For example, during M1 polarization, nitric oxide induced blunting of oxidative phosphorylation allows for increased ROS production via reverse electron transport (RET) at CI, CIII, and CIV [82]. Additionally, the cessation of electron generation necessitates the use of glycolytically generated ATP to drive F1F0 ATP transport of protons to prevent ΔΨm depolarization, increasing the ΔΨm and preventing the release of the pro-apoptosis signal cytochrome c to the cytosol [83]. Furthermore, mitochondria are recruited to the lysosome to assist in phagosome breakdown with ROS production via the TRAF6-ECSIT complex [84]. Metabolically, succinate accumulates during OXPHOS impairment, resulting in HIF1α stabilization, inflammasome activation, and IL-1β production [85]. Conversely, OXPHOS is necessary for IL-4 mediated polarization. Active flux through OXPHOS promotes cellular α-ketoglutarate (a-KG), which has been shown to suppress inflammatory gene expression. a-KG impairs activation of the NF-κB pathway, destabilizing HIF1α, and suppresses activation of Jmjd3 while promoting further fatty-acid oxidation to fuel OXPHOS [86,87]. Interestingly, M1 macrophage polarization via LPS+INF-γ results in reduced mitochondrial mass compared to M2 polarization via IL-4, potentially through incurred mitochondrial damage [88]. These observations suggest increased mitochondrial damage is associated with macrophage inflammatory activation. In contrast, during times of low glucose availability, upregulation of ATP production by OXPHOS may preserve inflammatory and immune functions [89,90].

## MITOCHONDRIAL DYNAMICS

Macrophage responsiveness is reliant upon mitochondria function. Damaged mitochondria may result in a compromised immune response, disturbed ROS production, and/or senescence [91–93]. Partially damaged mitochondria can undergo fission to remove dysfunctional components or fusion to buffer transient mitochondrial defects [94]. The central regulators of fission and fusion are DRP1 and OPA1, MFN1, and MFN2 respectively [95–99]. Mitochondrial fission serves to remove damaged mitochondria that may aberrantly generate ROS, while fusion also serves to preserve and share mtDNA throughout the network. Mitochondria dynamically undergo fission and fusion to remove dysfunctional mitochondria while preserving functional aspects.

**Figure 1.**
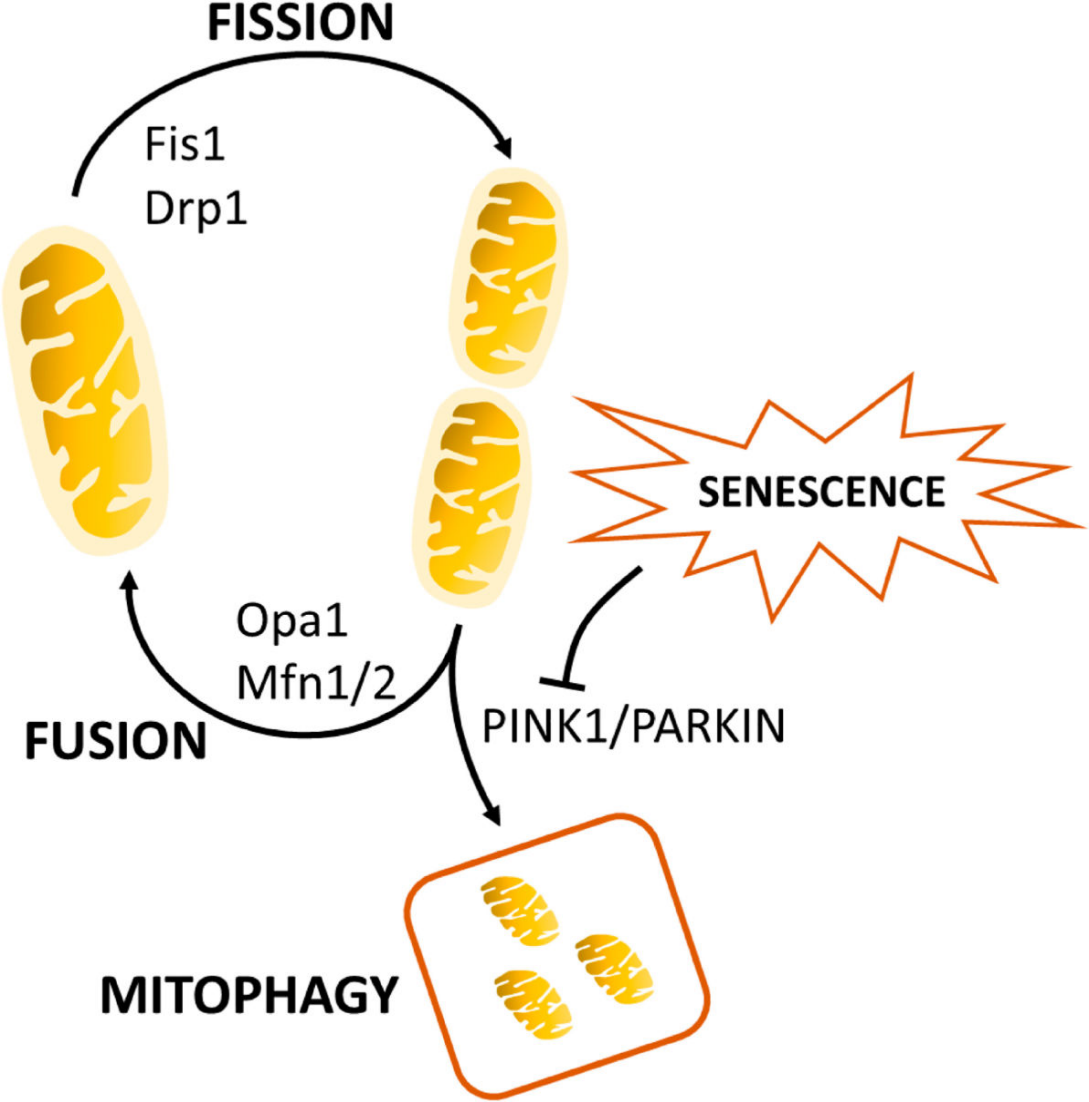
The mitochondrial lifecycle. Fused mitochondria undergo fission through Fis1. Individual mitochondria can undergo fusion through the actions of Drp1, Opa1, Mfn1, and Mfn2, or can be shuttled to the mitophagy pathway via PINK1 and Parkin. Cellular senescence inhibits mitophagy to induce mitochondrial dysfunction.

More severe mitochondrial damage may necessitate its removal. Mitophagy is the organelle-specific degradation of mitochondria that removes damaged or superfluous mitochondria. The most well-studied mitophagy pathway is ubiquitin-dependent PINK1-PRKN/PRK2. Briefly, increased ROS and loss of the ΔΨm results in PINK1 stabilization on the OMM, ubiquination, recruitment of E3 ligases PRKN/PRKN2 [100–104], and activation of ATGs [105]. Mitophagy may play a critical role in attenuating inflammatory responses. Endogenous and exogenous cellular danger signals such as ROS, TLR signaling, or mtDNA activation can serve to prime and activate the NLRP3 inflammasome and IL-1β secretion. Supporting this notion, inhibition of mitophagy proteins ATG15 or PRKN leads to increased IL-1β secretion following LPS stimulation [106–109]. Conversely, induction of mitophagy via ULK1 eliminates ΔΨm depolarization and mtROS to reduce caspase-1 activation and IL-1β secretion [110,111]. As such, mitophagy may be considered to control inflammation, as dysfunctional mitochondria are removed to minimize incidental inflammatory stimulation. As mitophagy is impaired with age, its dysregulation likely contributes to inflammaging, and this has been associated with several age-related diseases including cardiovascular disease and sarcopenia [112–114]. A schematic of mitochondrial dynamics is shown in Figure 1.

## INTER-ORGANELLE CROSSTALK

Mitochondrial dysfunction extends towards interactions with other organelles, so it is also important to consider their relationship during inflammaging. Mitochondria can migrate towards lysosomes for ion and metabolite transfer and to aid in inflammatory processes such as phagosome breakdown [115,116]. Chronic mitochondrial stress, which occurs during inflammaging, can impair lysosomal functions [117,118]. Lysosomal impairment leads to an accumulation of lipofuscin which can further dysregulate mitochondrial functions, including impaired mitophagy, increased ROS production, and reduced ATP generation [119]. Crosstalk between the ER and mitochondria also play an important role in aging. The ER is involved in protein synthesis and folding, and interaction between the ER and mitochondrial associated membranes regulates cellular Ca^2+^ [120,121]. ER stress triggers the unfolded protein response (UPR) to alleviate the protein burden and prevent misfolding, and chronic UPR activation by garbage in the inflammaging environment can lead to impaired proteostasis and acceleration of age-related diseases [122].

## MITOCHONDRIAL DYSFUNCTION AND CELLULAR SENESCENCE

Cellular senescence is a generally irreversible state of stable growth-arrest in proliferative cells which is resistant to apoptosis and is accompanied by phenotypic changes that contribute to aging. Senescence was originally demonstrated to occur to human fibroblasts in culture after repeated passaging [123], which is now known to be due to telomere attrition [124]. This has since been shown to occur in multiple cell types including in post-mitotic cells, as a result of exposure to various stressors [125,126]. Senescence does have beneficial roles as it can prevent the spread of (and stimulate immune cells to remove) malignant cells [127], and it is also involved in embryonic development and wound healing [128,129]. However, the number of senescent cells (SCs) accumulate with age in multiple tissues [126] and has been causally implicated in age-related dysfunction [130]. Senescence has thus been proposed to have evolved as a form of antagonistic pleiotropy, being beneficial to survival in young age, but detrimental in older age, with species-specific selection pressures driving a balance between tissue repair on the one hand and tumor suppression on the other [131].

SCs impart their effects through their senescence-associated secretory phenotype (SASP), which is characterized by an upregulation and secretion of proinflammatory cytokines, chemokines, exosomes, and other biological modulators which have autocrine, paracrine and systemic effects [132]. The primary role of the SASP within younger individuals may be to prevent the spread of damaged, senescent, or oncogenic cells by signaling to the immune system for clearance, but during aging, cellular damage accumulation may cause SC abundance to exceed the capacity for clearance by the immune system [127,133]. In conjunction, immune dysfunction caused by aging and senescence can reduce the ability of immune cells to clear SCs, further amplifying the accumulation of SCs with age [127]. There appears to be a threshold number of SCs above which age-related pathologies result, and this generally occurs around the ages of 60–70 years in humans [130]. SC accumulation promotes pathology on multiple levels. SCs contribute to inflammaging [134], can cause chronic damage to tissues and impair their normal physiological functions, and likely contribute to immune dysfunction [127].

During aging and senescence, the regulatory mechanisms governing mitochondrial quality are reduced. These changes are summarized in Table 1. Mitophagy and fission, as observed by a decrease in DRP1 and FIS1, are reduced during aging and mitochondria appear in hyper-fused states [135–137]. Senescent and aged cells display a decreased ΔΨm, increased proton leakage, aberrant ROS generation, and an increase in TCA intermediates [91,92,135,138]. These defects in cellular energy generation likely play a role in aberrant cytokine production and reduced immune competence. Furthermore, depletion of mitochondria via PINK1/Parkin induction eliminates the SASP [139]. While an extreme model that also resulted in cell cycle arrest, these data highlight a central role for mitochondria in cellular senescence. Thus, interventional strategies directed at restoring the mechanisms governing mitochondrial maintenance may serve as effective in combating the SASP and inflammaging. For instance, dysfunctional mitochondria in SCs were shown to drive formation of cytosolic chromatin fragments (CCFs) and the SASP via a ROS-JNK retrograde signaling pathway. Restoration of mitochondrial function via low-dose pharmaceutical class I and II histone deacetylase inhibitors (HDACi) were shown to suppress the SASP and formation of CCFs, while higher dosages were found to have senolytic activity [140]. A proposed mechanism for this effect is that the HDACi upregulate nuclear-encoded OXPHOS genes and suppress oxidative stress from ROS, although HDACi may also restore mitochondrial function by other means such as enhanced mitophagy [140]. Regardless, restoration of mitochondrial function through pharmacological or lifestyle interventions may present an effective strategy for reducing the harmful effects caused by SCs and may also serve as a preventative measure of SC accumulation with age.

## MACROPHAGE NAD BIOSYNTHESIS AND CONSUMPTION

NAD plays key roles in biological processes and has become of major interest in the aging field. Declining NAD levels with age have been documented in most tissue and cell types, including macrophages [141], and this is linked with aging and its associated diseases [142]. NAD is a coenzyme mediating many redox reactions crucial to metabolism and is also an essential cofactor for several NAD-consuming enzymes implicated with aging, including sirtuins (SIRTs), poly-ADP-ribose polymerases (PARPs), and CD38 [142]. NAD can be obtained from tryptophan via the de novo biosynthesis pathway, also called the kynurenine pathway (KP), nicotinic acid (NA) through the Preiss-Handler pathway, and recycling from nicotinamide (NAM) via the salvage pathway [143]. The NAD precursors nicotinamide mononucleotide (NMN) and nicotinamide riboside (NR), which is converted into NMN, can also contribute to NAD production through the salvage pathway and are increasingly being used as an exogenous method to raise NAD levels, as they have better bioavailability than NA [144,145]. NAD decline with age has been proposed to be caused by reduced synthesis, recycling, and/or increased consumption of it [146]. However, plasma levels of NAD precursors like NA and NAM were recently shown to remain stable with age, though plasma NAD levels were drastically reduced, which suggests NAD decline may be primarily due to an increase in the activity of NAD-consuming enzymes [147].

**Figure 2.**
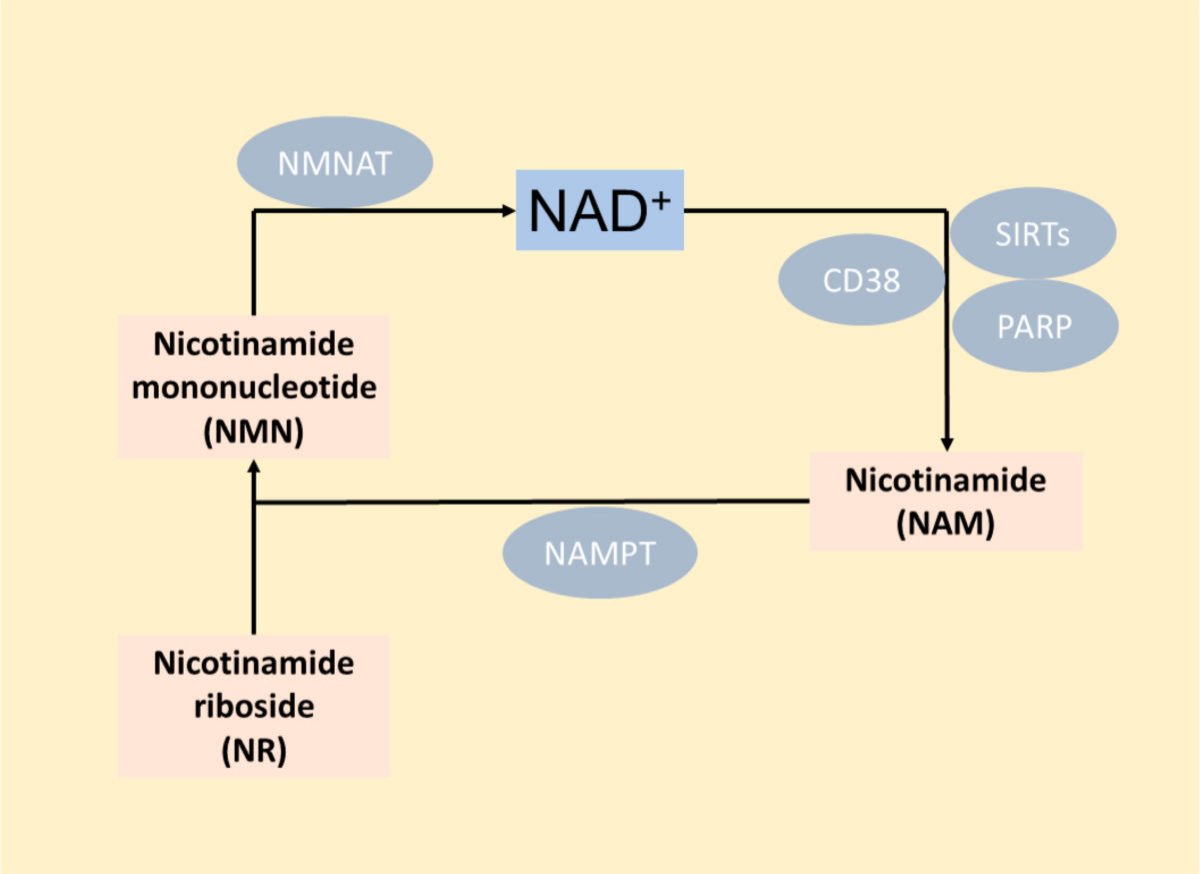
NAD salvage pathway.

The majority of intracellular NAD in most cell types is thought to be obtained via the salvage pathway [148], although the relative contribution of each pathway to NAD levels in macrophages is currently unknown. Further, recent studies have reported conflicting data as to the primary sources of NAD in macrophages, and the main NAD-consuming enzymes which cause it to decline [37,141,149]. It is likely dependent on the type of macrophage studied, whether it has been polarized to an inflammatory state, the organism it was derived from and its age, or a number of other factors. The majority of de novo NAD synthesis was shown to primarily occur in the liver, which excretes NAM for use in other tissues [148], although Minhas et al. recently showed that macrophages also rely on de novo NAD synthesis, and the activity of this pathway decreases with age and causes macrophage dysfunction [37]. The salvage pathway (Figure 2) appears to be the major contributor to NAD production in macrophages after inflammatory insults, and the rate limiting enzyme of this pathway, NAMPT, has been shown to be induced by TNFα, IL-1β, LPS, IFNγ, and hypoxia, all of which increase with age [141,149,150]. NAMPT is likely induced in inflammatory macrophages to keep up with NAD demand due to increased expression of the NAD-consuming enzyme CD38, which also degrades NMN and thus may reduce the effectiveness of NAD-replacement therapies [141,151].

While the salvage pathway is likely the primary contributor to NAD production in activated M1 macrophages, the de novo pathway may play a significant role during resting conditions. Minhas et al. recently demonstrated that the de novo pathway accounts for 40% of basal NAD production in human monocyte-derived macrophages (hMDMs). Additionally, they showed that inhibition of IDO1, which catalyzes the first step in the de novo pathway, altered mitochondrial morphology, suppressed OCR, and increased glycolytic activity [37]. Inhibition of QPRT, which is downstream of IDO1 in the de novo pathway, caused similar changes to mitochondrial morphology and metabolism, led to increased proinflammatory factors, and impaired phagocytosis. QPRT converts quinolinic acid into nicotinic acid mononucleotide (NAMN) which is ultimately converted into NAD. Aged hMDMs showed a significant decline in QPRT expression, de novo NAD synthesis, and SIRT3 activity, with increased polarization towards a proinflammatory state. Overexpressing QPRT, or supplementing with NMN, reversed these effects [37].

## AGE-RELATED NAD DECLINE: ROLE OF CD38 ACTIVATION IN MACROPHAGES

CD38 is a transmembrane protein involved in Ca^2+^ signaling and mobilization and mediates signal transduction, cell adhesion, activation, proliferation and differentiation, and has been found in nearly every cell type examined [152–154]. CD38 is necessary for effective immune responses, as CD38 deficient mice have increased susceptibility to infections [155]. CD38 is active both intra- and extracellularly and was originally thought to work primarily extracellularly, but recent evidence suggests the majority of CD38 activity is intracellular in macrophages, with its primary function being the generation of cyclic ADP-ribose and NAADP for Ca^2+^ regulation [141,153].

CD38 expression increases in multiple tissue types with age, significantly contributes to NAD decline, and may be caused exclusively by activation of tissue-resident macrophages from the SASP and inflammaging environment [141,151]. In particular, TNFα, IL-10, and IL-6, as well as numerous PAMP TLR ligands including LPS, were each individually shown by Covarrubias et al. to significantly upregulate CD38 expression in M1 polarized macrophages, but not M2 or M0 [141,154,155]. Macrophages cultured in media from senescent fibroblast or preadipocyte cells also markedly upregulated CD38, but they did not significantly upregulate other NAD-consuming enzymes such as PARPs or SIRTs [141]. The senescent fibroblasts and preadipocyte cells themselves did not show significant upregulation in CD38 expression. Further, intraperitoneal (IP) injection of the drug doxorubicin, which induces senescence, caused an accumulation of CD38^+^ macrophages in white adipose and liver tissue, with an increase in senescent markers and proinflammatory cytokines, similar to that observed with aging [141]. Indeed, tissue-resident macrophages of the liver (Kupffer cells) accumulate with age, express greater amounts of CD38, show more signs of senescence, and are skewed towards a proinflammatory polarization [141]. Other immune populations in the liver and hepatocytes showed low CD38 expression. Endothelial cells were the only other population in the liver other than Kupffer cells which highly expressed CD38, but they showed only a marginal increase in CD38 expression with age [141]. Therefore, this evidence suggests macrophages may be primarily responsible for the age-related increase in CD38 expression and NAD decline seen in tissues with aging.

NAD decline caused by CD38 activation likely contributes to age-related pathologies on multiple levels. Overexpression of CD38, but not SIRT1 or PARP1, was shown to have detrimental impacts on mitochondrial function and morphology, causing a dramatic decrease in total respiratory capacity, mitochondrial-driven ATP synthesis, NAD levels, and oxygen consumption rate (OCR) [151]. These defects may be due in part to reduced NAD availability for SIRTs, especially the mitochondrial protein SIRT3, which is essential for mitochondrial metabolism and function [151,156]. We previously showed that classical monocytes from older adults have reduced respiratory capacity [157] and hypothesize that declining NAD levels as a result of activation of CD38 by the SASP may be a key contributing factor. Interestingly, the first successful intervention reported to reduce epigenetic age in humans showed that diminishing CD38^+^ monocyte concentrations, as a result of the intervention, correlated with reduced epigenetic age, and they hypothesized that a subsequent increase in NAD tissue availability may have been largely responsible [158]. Although CD38 activity in monocytes is understudied, it is known that most classical and intermediate monocytes express it, as it plays an important role in extravasation of monocytes into tissues [155]. While a fewer percentage of nonclassical monocytes express CD38, a minority of them were shown to highly express it and were associated with inflammatory disease activity [155]. As nonclassical monocytes increase with aging [157], are more prone to senescence than the other subsets [159], and senescence has been shown to increase CD38 expression in macrophages, senescent nonclassical monocytes may also contribute to NAD decline seen with aging both in tissues and in circulation.

Besides being activated by the inflammaging environment, CD38 may also contribute to it, as overexpression promotes IL-1β, IL-6, IL-12, and glycolytic activity in hMDMs, and CD38 knockout (KO) mice have preserved mitochondrial function, SIRT3 activity, and were protected from NAD decline with age [155]. Although there is scant evidence on the interaction between CD38 and NF-κB, CD38 activation may increase NF-κB signaling. NF-κB is likely the primary transcription factor involved in the appearance of the SASP, and most of the proinflammatory genes expressed in senescent cells require it [132,160]. Therefore, it is of little surprise that NF-κB can activate CD38 [161], though recent evidence suggests CD38 may further amplify NF-κB signaling. CD38 KO mice were found to have greatly diminished NF-κB signaling in an autoimmune arthritic mouse model [162]. Further, inhibition of CD38 by the senolytic flavonoid quercetin was shown to reduce NF-κB signaling and M1 macrophage polarization in kidney and spleen tissue following IP LPS administration [154]. Since CD38 degrades NAD and NMN, inhibition with quercetin, when used in conjunction with NMN or NR therapy may be a viable method for increasing NAD levels, reducing proinflammatory macrophage polarization and senescence, and improving age-related pathologies.

As PARP1 and SIRT1 levels were found to decrease in several tissues with aging, CD38 is likely the main NAD-consuming enzyme contributing to age-related NAD decline [151], though the role PARPs play is inconclusive due to the abundance of contradictory findings in the literature. There is evidence for PARP1 both contributing to aging pathologies on the one hand by reducing NAD levels, and as a longevity-promoter on the other, and we recommend several review articles for more information [163,164]. PARP1 activity in macrophages specifically may contribute to inflammaging as it has been demonstrated to promote NF-κB and HMGB1 activity following LPS-stimulation [165,166]. Further, PARP1 inhibition has been shown to have anti-inflammatory effects [167]. While Covarrubias et al. reported no significant upregulation in PARP expression from senescent media, or following LPS administration in mice (though there was a trend upwards) [141], Cameron et al. found that LPS acutely caused ROS generation and DNA damage in BMDM, which stimulated PARP activity and led to NAMPT activation to keep up with NAD demand [37]. Like Covarrubias et al., they did not find an increase in enzymes of the Preiss-Handler or de novo pathway, and inhibiting NAMPT with FK866 led to a significant suppression in NAD levels in M1 macrophages, but not M0 or M2 [141,149]. Further, NAMPT inhibition diminished glycolytic activity and inflammatory mediators in M1 macrophages in vitro due to decreased GAPDH activity, which is NAD dependent [149]. This suggests NAD and NAMPT play critical roles during inflammatory activation in macrophages. The discrepancy between these two studies in PARP expression in M1 macrophages following LPS-stimulation may be due to the time point of analysis. It may be that early decreases in NAD macrophage concentrations after LPS-stimulation are due to oxidative stress and resulting PARP activation, whereas ongoing chronic depletion may be due to increased CD38 expression resulting from the inflammaging environment [141,149].

Table 2 contains an overview of major findings discussed above.

## CONCLUSION

**Figure 3.**
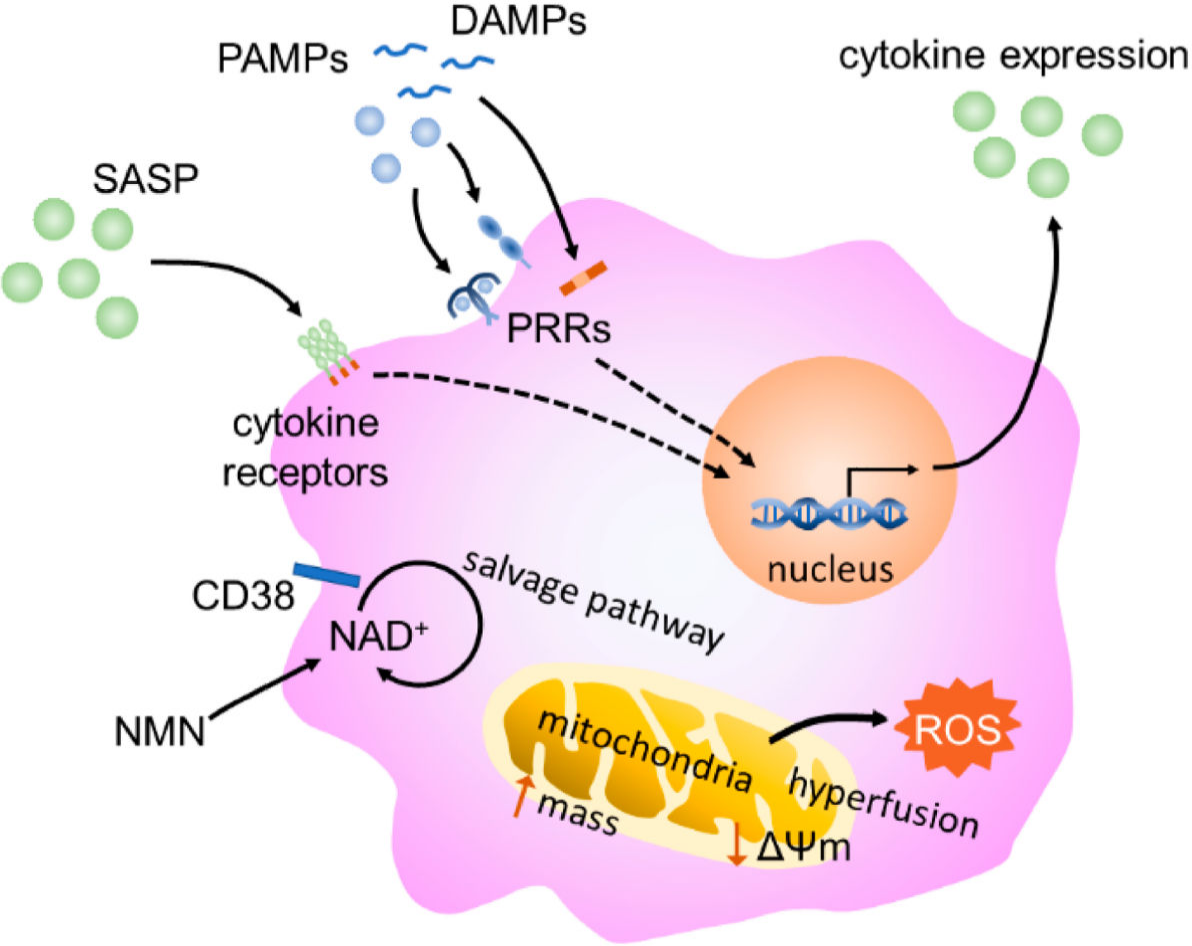
Schematic of an aging macrophage. Stimulation of the macrophage by SASP constituents, PAMPs, and DAMPs leads to intracellular signaling and propagation of the inflammatory state. Senescence-associated mitochondrial dysfunction, including increased ROS production and mitochondrial mass, mitochondrial hyperfusion, and decreased membrane potential, may also play a role in immunometabolic changes in aging macrophages. Finally, CD38 expression increases consumption of NAD through the salvage pathway, leading to lower tissue levels of NAD.

Figure 3 outlines the major themes of this review. Accumulation of damage over time increases the number of DAMPs and PAMPs in circulation which seem to drive metabolic-dependent epigenetic changes that alter macrophage functions during aging. Among these alterations is a heightened basal state of inflammation, diminished or hyperactive inflammatory responses, and impaired effector functions. A significant number of DAMPs are derived from malfunctioning mitochondria. With age mitochondrial dynamics, mitophagy, and inter-organelle crosstalk are impaired leading to enhanced oxidative stress, mtDNA excretion, altered metabolism, and impaired proteostasis. Agie-related NAD decline plays a major role in mitochondrial dysfunction and new evidence suggests this decline may be largely due to an upregulation of CD38 in tissue-resident macrophages caused by the SASP, PAMPs, and other proinflammatory factors in the inflammaging environment. CD38 activation in monocytes may also play a significant role in NAD decline, but other immune cells such as lymphocytes and neutrophils do not seem to be a major contributor. Recently, there has been an ever-increasing amount of evidence demonstrating NAD supplementation to be effective in protecting against age-related pathologies [168]. The results reviewed here indicate that inhibition of CD38 in conjunction with NAD supplementation may be more effective than NAD supplementation alone as CD38 degrades both NAD and NMN.

## Figures and Tables

**Table 1.** Summary of age-related mitochondrial dysfunction.

Phenomenon	Aging Effect
mtDNA release	↑
Mitophagy	↓
Fission	↓
Fusion	↑
∆ψm	↓
Proton leak	↑
mtROS production	↑

∆ψm: mitochondrial membrane potential. mtDNA: mitochondrial DNA. mtROS: mitochondrial reactive oxygen species.

**Table 2.** Macrophages, CD38, and nad metabolism.

Species	Cell Type	Stimulus/Condition	Effect	Reference
C57BL/6J mice	BMDM	Cytokines, TLR ligands LPS	↑ CD38	[141]
C57BL/6J mice	WAT mϕ	LPS	↑ CD38 ↑ TNFα, IL-1β ↑ mϕ	[141]
C57BL/6J mice	BMDM	Senescent media	↑ CD38	[141]
C57BL/6J mice	Liver and WAT mϕ	Doxorubicin	↑ CD38^+^ mϕ	[141]
C57BL/6J mice	Kupffer cells	Aging (25 months old compared to 6 months)	↑ CD38, p21, NAMPT, IL-1β, IL-18, CCL2, HMGB1 ↑ mϕ ↑ M1 polarization	[141]
Human (cell line)	HEK293T	CD38 overexpression	↓ mitochondrial respiratory capacity ↓ NAD Abnormal mitochondrial morphology	[151]
C57BL/6 mice	Isolated liver mitochondria	CD38 KO vs WT	↑ respiratory rate ↑ NAD and NAD/NADH ↑ SIRT3	[151]
Human	Monocytes	Epigenetic age reversal intervention	↓ CD38^+^ monocytes	[158]
Human (cell line)	THP-1, U937	LPS + IFNγ	↑ CD38 vs M0 or M2	[155]
Human (cell line)	THP-1, U937	CD38 inhibition via apigenin or rhein in M1 (LPS + IFNγ)	↓ IL-6, IL-12p40	[155]
C57BL/6 mice	BMDC	CD38 KO vs WT with collagen-induced arthritis	↓ phospho-NFκB ↓ IL-1β, IL-4, IL-10	[162]
Mouse (cell line)	RAW 264.7	LPS	CD38 activation	[154]
Mouse (cell line)	RAW 264.7	LPS stimulation with CD38 inhibition via quercetin	↓ phospho-NFκB ↓ M1 polarization	[154]
C57BL/6 mice	Kidney mϕ	LPS stimulation with CD38 inhibition via quercetin	↓ mϕ accumulation ↓ CCL2 and TNFα ↓ M1 polarization ↓ phospho-NF-κB	[154]

BMDC: bone marrow-derived dendritic cells; BMDM: bone marrow-derived macrophages, CD: cluster of differentiation; IFN: interferon; IL: interleukin; KO: knockout; LPS: lipopolysaccharide; WAT: white adipose tissue; WT: wildtype.

## ABBREVIATIONS

ΔΨm: mitochondrial membrane potential
BMDM: bone marrow-derived macrophage
DAMP: damage-associated molecular pattern
ER: endoplasmic reticulum
ETC: electron transport chain
FAO: fatty acid oxidation
HDAC: histone deacetylase
hMDM: human monocyte-derived macrophage
IFN: interferon
IL: interleukin
IMM: inner mitochondrial membrane
KO: knockout
KP: kynurenine pathway
LPS: lipopolysaccharide
mtDNA: mitochondrial DNA
NA: nicotinic acid
NAD: nicotinamide adenine dinucleotide
NAM: nicotinamide
NLR: NOD-like receptor
NMN: nicotinamide mononucleotide
NR: nicotinamide riboside
OMM: outer mitochondrial membrane
OXPHOS: oxidative phosphorylation
PAMP: pathogen-associated molecular pattern
PARP: poly-ADP-ribose polymerase
PRR: pattern recognition receptor
RAGE: receptor for advanced glycation end products
RET: reverse electron transport
ROS: reactive oxygen species
SASP: senescence-associated secretory phenotype
SC: senescent cell
SIRT: sirtuin
TCA: citric acid cycle
TFAM: mitochondrial transcription factor A
TNF: tumor necrosis factor
TLR: toll-like receptor
